# Habituation After Deep Brain Stimulation in Tremor Syndromes: Prevalence, Risk Factors and Long-Term Outcomes

**DOI:** 10.3389/fneur.2021.696950

**Published:** 2021-08-03

**Authors:** James Peters, Stephen Tisch

**Affiliations:** ^1^Department of Neurology, St Vincent's Hospital, Sydney, NSW, Australia; ^2^School of Medicine, University of New South Wales, Sydney, NSW, Australia

**Keywords:** tremor, deep brain stimulation, habituation, dystonic tremor, essential tremor, Parkinosn's disease

## Abstract

Deep brain stimulation (DBS) of the thalamus is an effective treatment for medically refractory essential, dystonic and Parkinson's tremor. It may also provide benefit in less common tremor syndromes including, post-traumatic, cerebellar, Holmes, neuropathic and orthostatic tremor. The long-term benefit of DBS in essential and dystonic tremor (ET/DT) often wanes over time, a phenomena referred to as stimulation “tolerance” or “habituation”. While habituation is generally accepted to exist, it remains controversial. Attempts to quantify habituation have revealed conflicting reports. Placebo effects, loss of micro-lesional effect, disease related progression, suboptimal stimulation and stimulation related side-effects may all contribute to the loss of sustained long-term therapeutic effect. Habituation often presents as substantial loss of initial DBS benefit occurring as early as a few months after initial stimulation; a complex and feared issue when faced in the setting of optimal electrode placement. Simply increasing stimulation current tends only to propagate tremor severity and induce stimulation related side effects. The report by Paschen and colleagues of worsening tremor scores in the “On” vs. “Off” stimulation state over time, even after accounting for “rebound” tremor, supports the concept of habituation. However, these findings have not been consistent across all studies. Chronic high intensity stimulation has been hypothesized to induce detrimental plastic effects on tremor networks, with some lines of evidence that DT and ET may be more susceptible than Parkinson's tremor to habituation. However, Tsuboi and colleague's recent longitudinal follow-up in dystonic and “pure” essential tremor suggests otherwise. Alternatively, post-mortem findings support a biological adaption to stimulation. The prevalence and etiology of habituation is still not fully understood and management remains difficult. A recent study reported that alternating thalamic stimulation parameters at weekly intervals provided improved stability of tremor control consistent with reduced habituation. In this article the available evidence for habituation after DBS for tremor syndromes is reviewed; including its prevalence, time-course, possible mechanisms; along with expected long-term outcomes for tremor and factors that may assist in predicting, preventing and managing habituation.

## Introduction

Tremor is an involuntary, rhythmic, oscillatory movement of a body part (1), with an estimated prevalence of 14.5% in the general adult population (2). The spectrum of tremor extends from the enhanced physiological postural tremor, often only noticeable during states of fatigue and heightened anxiety, to persistent pathological syndromes including essential tremor (ET), where currently available medications are moderately effective at best. For the severe end of the spectrum of tremor syndromes, functional neurosurgical techniques and neuroradiological procedures exists. These have evolved since Cooper (3) made the unintentional observation nearly 70 years ago, that destruction of a portion of the globus pallidus suppressed tremor of a patient with Parkinson's Disease (PD). Stereotactic lesional surgery mainly targeting the ventrolateral thalamus and posterior subthalamic white matter was used since the 1950's. During this period it was discovered that intraoperative high frequency electrical stimulation would suppress tremor and was used to verify the target region prior to thermal abalation (4, 5). These pioneering stereotactic interventions paved the way for the first cases of deep brain stimulation (DBS) performed by Cooper et al. (6). Motivated by a desire to avoid the frequent dysarthria observed following bilateral radiofrequency thalamotomy, DBS of the thalamic ventral intermediate nucleus (VIM) was revisited by Benabid in 1987 for second-side treatment of tremor and popularized following their 1991 publication of a series of 26 PD and six essential tremor (ET) patients treated with VIM DBS who reported to have a sustained tremor response over a 13-month median follow-up period (7). Subsequently large studies confirmed the effectiveness of VIM DBS for ET and PD (8, 9).

DBS remains the most common surgical procedure for medication-refractory tremor. However, the long-term benefits of therapy, particularly in ET, are often observed to wane over time, in a variable, unpredictable pattern. A phenomenon of “tolerance” was first described by Benabid et al. in a series of 80 tremor-dominant PD and 20 ET patients, with either uni- or bi-lateral VIM stimulation (10). Regular increase in stimulation to alleviate tremor was required to a final threshold that could no longer be increased due to the induction of side effects. “Tolerance” was associated with eventual loss of functional benefit and was more commonly observed with those with action tremor, in severe syndromes, with higher stimulation intensity, and where continuous 24-h stimulation had been adopted (10). The phenomenon of “tolerance” has continued to be observed in clinical practice and is now usually referred to as “habituation” (11). Attempts to characterize and quantity habituation have revealed conflicting reports in the medical literature and remain the subject of debate.

In this narrative review article, the available evidence for habituation after DBS for tremor syndromes is reviewed to reappraise perceptions of expected long term outcomes and factors that may assist in predicting this phenomenon. We also provide some information on possible pathophysiological mechanisms underlying ‘habituation' and approaches to its management.

## Habituation Definition

Habituation in the context of benefit from DBS was first mentioned in the medical literature by Benabid et al. and described as “tolerance”. It was hypothesized a progressively decreased biological response (habituation) of the neuronal network to be a possible mechanism for the phenomena of “tolerance” (10). Recently the term habituation has been proposed to replace “tolerance”, defined by Fasano and Helmich to be the rapid vanishing of DBS efficacy after programming (11). This definition of habituation can be expanded to include delayed, progressive loss of therapeutic benefit for tremor after DBS, in line with the original concept of “tolerance” due to “*decreased biological response (habituation) of the neuronal network”* as described by Benabid et al. (10).

Authors have attempted to study habituation in the context of progressive loss of DBS benefit with particular attention given to differentiating progression from the natural history of disease. We agree in theory that comparing the tremor severity in the “off” state at two different time points, after allowing for rebound, represent disease progression; whereas tremor severity in the “on” state is determined by both disease progression and the stimulation effect. The difference (delta) between the on-off state, when compared over time, has been assumed by authors to be a measure of changing stimulation over time and attributed to habituation (12, 13). This is based on the premise that over time, other variables, specifically lead location and optimization of programming remain constant; but further, alternative mechanisms are not contributing or causing the phenomena that has been labeled habituation. Given these provisions, we will proceed on the operational hypothesis, reflected by the change in delta over time, from the definition of habituation known previously as “tolerance”; to be the loss of benefit from electrode reprogramming over time in the setting of optimal electrode placement and programming not explained by disease progression of the tremor syndrome. Habituation should not be explained by loss of micro-lesional implant effect or expected progression due to the natural history of the tremor syndrome. In line with the concept of “rapid vanishing of effect” habituation also refers to temporary improvement in tremor severity following increasing electrical field strength or contact adjustment, followed by subsequent paradoxical worsening.

Although this definition is useful conceptually, determining if an individual patient is experiencing habituation after tremor DBS remains very difficult because of the following; Firstly, there is no absolute agreed definition of what constitutes optimal lead placement; more troubling though, is the fact not all DBS leads placed within the optimal 2 mm radius of the intended target have a concordant clinical response (14). Secondly, optimal DBS programming is highly operator dependent as evidenced by significant clinical improvements achieved after expert reprogramming (15). Lastly, progression of the underlying tremor syndrome as part of the natural history of the disease must be subtracted from any apportionment of habituation, in itself a very difficult distinction, highlighting the inherent complexity and uncertainties surrounding this topic.

## Does “Habituation” Really Exist?

### Loss of Benefit Over Time

Habituation has most commonly been associated with ET, possibly reflecting the experience of clinicians in practice. Over 20 studies have been published looking at the long-term clinical efficacy of DBS in this condition; most commonly involving uni-or bilateral VIM stimulation (12, 13, 15–32). When looking at studies with a greater than 3-year follow-up, the long-term effect compared to baseline, ranges from 31.2–88.4% improvement (11, 13). The less traditional target, posterior subthalamic area (PSA)/caudal zona incerta (cZi), in comparison has relatively few follow-up studies; but with a similar range of effect size from baseline: 33–76% improvement (30, 33, 34). Some studies have suggested that the PSA/cZI may be less prone to habituation; however, no superiority has ever been clearly established (33–35). Despite this persistent improvement from DBS in the long-term, the majority of studies have shown that the effect diminishes over time.

**Figure 1:**
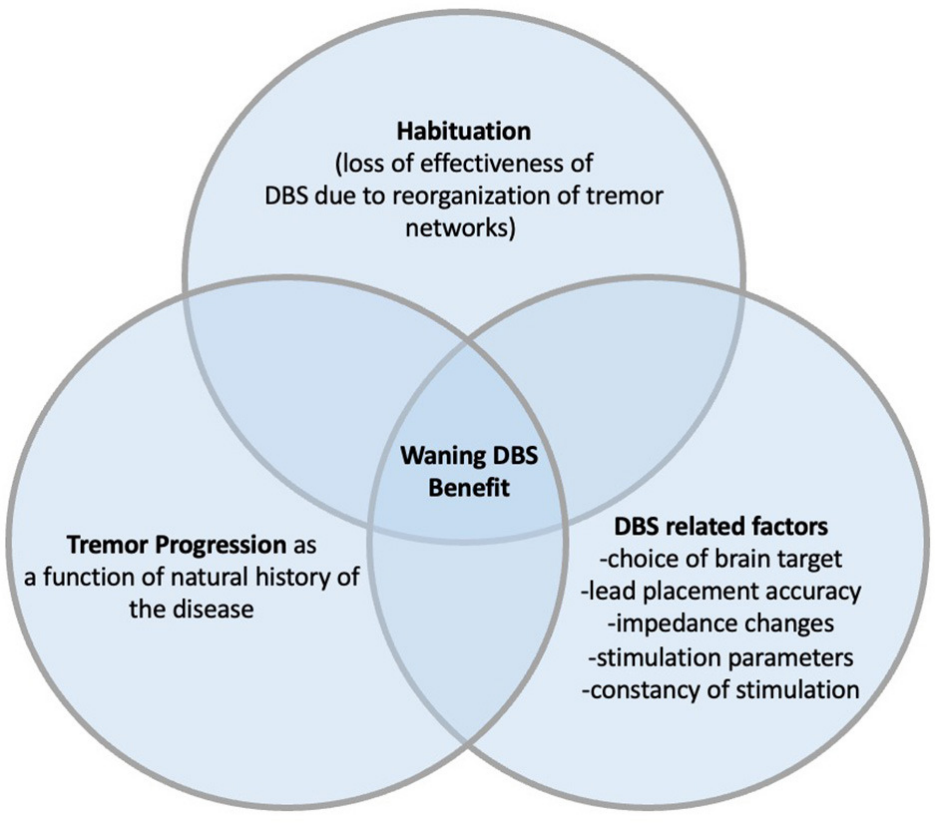
Factors contributing to decline in clinical benefit of tremor after DBS.

A recent systematic review and meta-analysis by Lu et al., included 26 studies with 439 patients, looked at potential outcome predictors following VIM DBS in ET. The pooled treatment effect was 60.3% improvement in objective Tremor Rating Scale (TRS) scores at 20 months (+/– 17.3). Correlation with outcome was seen only with pre-operative TRS scores and follow-up time; both negatively correlated with the clinical outcome (36). It had previously been reported that pre-operative cerebellar dysfunction was a risk factor for the development of early “tolerance” (37). Natural disease progression and habituation have been proposed as the most plausible factors contributing to VIM-DBS treatment declining overtime (17, 23, 27, 31, 33). Despite the absence of consensus guide lines, electrode placement beyond a 2–3 mm radius of an intended target have been associated with suboptimal tremor control and can be a correctable cause of DBS “failure” (37, 38). Further, in cases of suboptimal clinical benefit, DBS lead adjustment of only a few millimeters can have a meaningful benefit (39). Other possible co-contributing factors include incorrect pre-operative diagnosis (14), loss of microthalamotomy (9) and increased impedance of brain tissue over time (10) (Figure 1). However, effects of varying tissue impedance are minimized by constant current DBS systems now more widely used.

### Short-Term Habituation

Continuous increase in DBS stimulation parameters, followed by a temporary improvement in tremor severity but an ultimate paradoxical worsening is the hallmark clinical observation in habituation. This was first demonstrated in the short-term by Barbe et al. in patients treated with VIM DBS for ET. After optimization of stimulation parameters, patients were followed and reassessed at 10-weeks. Statistically significant improvement in TRS hemi-body scores compared to baseline was observed after optimization; but at 10-weeks this stimulation effect was remarkable weaker, abolishing the immediate effect compared to baseline (40). Furthermore, of the 21 patients who agreed to follow-up after initial stimulation changes, only 16 completed the 10-week assessment, with two patients dropping out due to unacceptable worsening of the tremor syndrome. Adaption of the pathological tremor networks to the new DBS interface was proposed, clinically seen as paradoxical worsening of tremor, referred to as “habitation” by the authors.

### Long Term Habituation or Disease Progression?

Separating natural disease progression and habituation in the context of gradual loss of DBS benefit overtime is difficult. In theory, comparing the “off” stimulation tremor severity at two different time points should only represent progression secondary to disease. While tremor severity in the “on” stimulation state over similar time points should reflect both disease progression and the stimulation effect (12, 13). Delta, the difference between the “off” and “on” state, when compared overtime, appears our best measure of any changing stimulation effect, and possible habituation. Of all the long-term DBS follow-up studies in ET, only seven (12, 17–19, 29, 32, 33) have “off/on” data at more than one defined time point, that allows the analysis of change in delta overtime, and the possible detection of habituation (Table 1). Further, the most recent study by Paschen et al. have calculated the difference in TRS score in both “off/on” states compared to baseline, allowing for statistical separation of disease progression and habituation (13).

**Table 1:** The change in deep brain stimulation effect in long term studies of Essential Tremor.

**Reference, Study type**	**Patients**	**Syndrome**	**Mean follow-up**	**DBS target**	**Uni/bilateral stimulation**	**Off assessment time^**∧**^**	Outcome^*^	**Exclusion & other**
Rehncrona et al. (17), prospective	*N* = 25 Exclusion = 14	ET and PD	ET: 6.5 yrs PD: 6.6 yrs	VIM	ET: 17/2 PD: 19/0	2-year: 4-hours 6–7 years: 1-hour	ET 2-year delta: 49% 6–7-year delta: 47% Loss of benefit: 4% PD 2-year delta: 77% 6–7-year delta: 54.5% Loss of benefit: 29%	ET *N* = 6 (3 dead, 1 refused, 1 lost, 1 battery life end) PD *N* = 8 (4 dead, 2 refused, 2 lost)
Sydow et al. (18), prospective	*N* = 19 Exclusion = 7	ET	6.54 yrs	VIM	Baseline: 15/4 6-years: 12/7	UN	1-year delta: 45.6% 6-year delta: 46.3% Gain of benefit: 1%	*N* = 7 (1 stopped due to SE, 3 dead, 1 refused, 1 lost, 1 battery life end)
Blomstedt et al. (21), prospective	*N* = 19 Exclusion = 8	ET	7.17 yrs	VIM	UN	UN	Initial (mean 13 months): delta: 52% Final (86 months) delta: 30% Loss of benefit: 42%	*N* = 8 (3 diagnosis revised, 4 died, 1 lost)
Favilla et al. (12), retrospective	*N* = 28 Controls = 21 Excluded = 41	ET	>36 months	VIM	19/9	30 mins	Unilateral 6 months delta: 27% 36 months delta: 26% Loss of benefit: 4% Bilateral 6 months delta: 63% 36 months delta: 37% Loss of benefit: 41%	*N* = 41 (10 prior DBS outside facility, 4 stimulator revision, 13 lost, 11 follow-up <24months)
Fytagoridis et al. (33), prospective	*N* = 18	ET	4.04 yrs	cZI	16/2	DBS deactivated overnight	1-year delta: 54.5% 3-5-year delta: 51.4% Loss of benefit: 6%	-
Cury et al. (29), retrospective	*N* = 98	ET, PD & DT	ET: 8.1 yrs PD: 10.2 yrs DT: 10.8 yrs	VIM	ET = 35/3 PD = 24/30 DT = 2/4	60 mins	PD Bilateral 1-year delta:73% 11–15 year delta: 69% 16–21 years delta: 60% Loss of benefit: 18% Unilateral 1-year delta: 67% 11–16 years delta: 58% 16–21 years delta: 63% Loss of benefit: 6% ET year delta: 66% >10 years delta: 48% Loss of benefit: 27% DT Delta: UN	DT 4/6 received GPi DBS after VIM DBS, 3 due to lack of efficacy and intolerable side effects
Tsubio et al. (32), retrospective	*N* = 124 Exclusion = 40	ET & DT	ET: 3.5 yrs DT: 3.4 yrs	VIM	ET: 72/25 DT: 17/9	At least 30 mins	ET 6-month delta: 50% 1–year delta: 42% 2–3 year delta: 37% 4–5 year delta: 38% 6year delta: 34% Loss of benefit: 32% DT 6-month delta: 42% 1–year delta: 45% 2–3 year delta: 41% 4–5 year delta: 8% 6–year delta: 42% No loss or gain of benefit	*N* = 40 (24 alternative diagnosis and 16 lack of assessments)

*cZI, caudal zona incerta; DBS, deep brain stimulation; DT, dystonic tremor; ET, essential tremor; GPi, globus pallidus internus; N, number; PD, Parkinson Disease; PSA, posterior subthalamic area; SE, side effect; UN, unknown; VIM, ventralis intermedis nucleus of thalamus*.

∧ *Time after DBS was switched off*.

* *Statistical significance of delta at each time point or change in delta overtime (loss or gain of benefit) were not able to be verified*.

In the seven long-term DBS studies in ET that data is available to determine the percentage of delta change over time, loss of stimulation benefit was seen in all but one study (17). The effect lost over time, as a percentage of the stimulation effect on the first assessment compared to the last assessment, ranged from 4–42% (Table 1). However, owing to the design of the studies, the statistical significance of these changes remains unclear in all but one study, where subgroup analysis revealed the loss of effect to be not significant (*p* > 0.05). The target of DBS stimulation was the VIM in six (12, 17–19, 29, 33) and the PSA in one (32) of these studies. In five of the studies the exclusion and drop-out rate ranged from 24–59%, with drop-outs often including patients with progressive tremor severity, unacceptable side-effects or even stimulation revision. This could possibly lead to an underestimation of the loss of stimulation benefit over time. During follow-up, stimulation parameters increased across three studies, remained unchanged or statistical insignificant across two studies and were not reported in two.

Limitations beyond the high drop-out rate exists across all these studies. Only one group look at the relationship between lead location and tremor response, despite it being a known cause for chronic loss of DBS benefit over time. Cury et al. reported that stimulation to the caudal part of the right VIM was associated with a worse tremor result at 1-year but how this related to the change in delta overtime and habituation is unknown (29). Favilla et al. who concluded that disease progression is the most likely explanation for worsening tremor after DBS (12), failed to assess the change in response to stimulation over time in patients whose tremor was either stable or improved in the “off” state. Assessment including this cohort reveals a 4 and 41% loss of stimulation benefit between 6 and 36 months in the unilateral and bilateral stimulated groups respectively. Paschen et al. attributed 13% of the worsening “stim-on” to habituation (13). However, overestimation to disease progression is likely to have occurred after the mean monthly worsening of the TRS scores in the “off” and “on” state were calculated from different time points. In which, part of the decline in the “off” state is likely due to the loss of microthalamotomy that is not reflected in the TRS stim-on scores. In the most recent study by Tsuboi et al. delta values need to be interpreted with caution as not all patients were assessed in the “off” state at each separate time point (33).

### Emerging Ataxia and Rebound Tremor as Biomarkers for Habituation?

The relationship between stimulation-induced ataxia and habituation is unclear. The two entities are often addressed independently despite the clinical observation that some patients develop a progressive ataxic cerebellar syndrome after bilateral VIM DBS for tremor when the stimulation intensity is increased (41, 42). This syndrome may be characterized by dysarthria, gait unsteadiness, limb incoordination and tremor of a different quality to the original tremor syndrome and often worse (43). DBS induced ataxia is not rare occurring in a third of patients in one large series (44). The cerebellar signs may dissipate if DBS is stopped and allowed to wash-out over several days, usually revealing the original tremor syndrome which may be more manageable than the DBS induced ataxic syndrome (43, 45). Induction and reversibility of the cerebellar dysfunction in these patients implies long-term aberrant plasticity within cerebellar networks. This clinical observation has been supported by functional imaging that has demonstrated stimulation induced hypermetabolism in the cerebellar nodule, exclusive to those with this syndrome, associated with stimulation particularly in the sub-thalamic white matter, the effect linked to antidromic stimulation of cerebello-thalamic fibers (45). Furthermore, a post-mortem study identified preservation of climbing and parallel cerebellum input fibers exclusive to those who had undergone DBS (46). Intriguingly, VIM DBS has also been shown to improve gait and limb ataxia in ET patients, independent of tremor-suppressing effects, provided the stimulation intensity is not excessive, at which point ataxia is worsened (41, 42). These data point to dual contribution of both stimulation proximity to cerebellar outflow tracts and intensity on the development of ataxia after VIM DBS.

Another phenomenon is rebound tremor where tremor severity is much worse than the pre-operative baseline immediately after DBS is switched off (10, 42, 45). This phenomenon has been strongly associated with the stimulation induced ataxic cerebellar syndrome, in which less tremor suppression benefit from stimulation was also observed (45). However, there has not been a clear association between rebound tremor and habituation. Nevertheless, switching the DBS off is a regular occurrence and many DBS groups advise patients to switch their device off at night in an effort to avoid habituation; although controlled studies to confirm this hypothesis are so far lacking. Not infrequently patients with waning tremor benefit and habituation describe more marked rebound tremor with the device off, such that some may become incapable of switching their device off at night owing to unacceptable worsening of tremor (43, 47).

### Deep Brain Stimulation and Dystonia–An Insight for Habituation?

DBS of the globus pallidus internus (GPi) is an effective treatment for primary dystonia (48). However, unlike other neurological conditions, maximal clinical benefit can take weeks to months (49). This often occurs with similar stimulation parameters, in the absence of an abrupt but monotonic improvement in dystonia (50). Longitudinal neurophysiological examinations have provided mechanistic insights into excessive muscle activity and overflow characteristics. After GPi DBS a quick absence of enhance per-operative plasticity is seen but the normalization of cortical inhibition takes months to achieve, following a similar time course to the clinical response (51). Further, these physiological changes do not immediately ablate after stopping the stimulation (52). The long-term effects on the cortical circuitry in dystonia are positive, but negative examples, in the form of emergent dystonia, after lesional and DBS surgery of the thalamus have been reported (53). Conceptually, habituation is likely a form of neural reorganization in a negative sense, with many similarities to the changes we observe after GPi DBS for dystonia.

## Habituation and Other Tremor Syndromes

### Parkinson's Disease Tremor

DBS for the management of PD is a well-established treatment (54–60). It has been shown to be superior to medical therapy in the early (54) through to advanced disease stages (60). The subthalamic nucleus (STN), GPi and VIM have all been shown to be effective DBS targets for tremor suppression (61, 62). Despite the initial reports of habituation involving a cohort predominantly of PD patients (10); little has been published in the literature since. Rechrona et al. demonstrated a 29% loss of effect with VIM stimulation over a 4–5-year follow-up period (17). More recently, Cury et al. showed up to an 18% loss of benefit with the same target over a duration of 16–21 years (29). However, other long-term follow-up studies have not demonstrated a similar waning benefit of DBS commonly associated with ET (61–65). In early-stage PD, STN DBS has been shown to slow the progression of rest tremor and provide long-term symptomatic benefit compared to standard medical care (54). Collectively these data may suggest that habituation is less likely to occur in PD tremor and less likely with DBS targets other than VIM.

### Dystonic Tremor

In comparison to ET and PD the long-term effectiveness of DBS for dystonic tremor is not as well established (66). Many studies have reported on the effectiveness of GPi DBS for dystonia without reporting on tremor outcomes (67). The thalamus, commonly the VIM, is the predominant target in DT but alternative or tandem targets (GPi or STN) are often used when dystonic symptoms are more problematic (68). In a systematic review, improvement in TRS motor scores from baseline was approximately 40–50% (66). Recent studies by Tsuboi et al. and Cury et al. reported tremor suppression benefit was not significant at greater than 5–6 years after implantation (29, 32). Moreover, four of six patients in the study by Cury et al. received additional GPi stimulation due to lack of efficacy or intolerable side effects (29). Others have report the similar need to proceed to an alternative DBS target to manage persistent or emergent dystonia and/or tremor after an initial single target DBS implantation (68, 69). The delta change over time could only be assessed in the Tsuboi et al. (32) cohort, where a loss of benefit was seen at the majority of time points (Table 1). The outlying result at greater than 6 years was considered non-significant by the authors (32). It has been hypothesized that these observations represent habituation (32). There is growing evidence dystonia results from widespread multi-level network dysfunction (70), involving the basal ganglia, cerebellum and excessive motor cortical plasticity, with evidence long-term network modification after GPi DBS (71), however the long-term effects of thalamic DBS on these networks in dystonia is unknown.

### Uncommon Tremor Syndromes

Apart from one randomized clinical trial in multiple sclerosis (MS)-associated tremor (72), experience with uncommon tremor syndromes; Holmes' tremor (HT) (73), orthostatic tremor (OT) (74), neuropathy-associated tremor and fragile X-associated tremor/ataxia syndrome (75); come from case reports and small case series. Habituation has been reported in neuropathic tremor from demyelinating neuropathy treated with VIM DBS, worse than a comparison ET group (76). Bi-or-unilateral VIM stimulation is the commonest modality of treatment used irrespective of tremor syndrome. Other targets of stimulation used independently or as an adjacent to the VIM included: cZi, GPi, PSA, STN, Ventro-lateral (VL), Ventralis oralis anterior or Ventralis oralis posterior nuclei (VOA/VOP) (75). Data suggests that DBS might be useful for these uncommon syndromes; but both the rarity of these conditions and heterogeneity makes the nature and magnitude of any effect uncertain (75). Furthermore, the same must be said for the development of unwanted events to stimulation including habituation.

## Factors That May Predispose to Habituation

### Does Habituation Differ Between Tremor Subtypes?

Attempting to identify factors that predisposed to habituation, a phenomenon hard to define and even more difficult to study, should be done with caution. However, the underlying disease seems to be an important factor in predicting long-term outcomes (29, 32). DT appears to be the tremor syndrome least responsive to DBS in the long-term and potentially the most susceptible to habituation (29, 32). Although Tsubio et al. reported comparable long-term tremor suppression results between DT and ET in VIM DBS, loss of stimulation benefit at greater than 6-years was only present in the DT cohort. Furthermore, improvement in activities of daily living tended to be greater in the ET cohort (32). Cury et al. have demonstrated a similar loss of tremor suppression benefit in DT compared with both ET and PD (29). This may reflect disease progression or emergent dystonia (10, 53) rather than habituation (32). Combined VIM and GPi DBS (77) could potentially alleviate some of these issues but long-term follow-up studies are required.

There is evidence to suggest habituation is less common in PD tremor treated with DBS. In the long-term comparison study of thalamic DBS in PD, ET and DT; greatest stimulation benefit was seen with PD tremor (Table 1) (29). In non-comparative studies of VIM, STN and GPi DBS; more stable consistent response to stimulation have been demonstrate (61–65). In some of these studies up to 50% of the cohort experience complete absence of rest tremor (62–65). One interesting observation after both VIM and STN DBS, tremor in the off-medication-off-stimulation condition is often less severe than the off-medication baseline state. This is surprising despite the knowledge that PD tremor doesn't necessarily get worse over time. Structural or neurochemical change leading to the improvement of tremor has been suggested; a persistent micro-thalamotomy effect or residual effect from electrical stimulation have not been excluded (64). This evidence would argue against habituation in PD tremor.

### Does Habituation Depend on DBS Target?

The DBS target for tremor suppression is of particular interest in view of the progressive loss of tremor benefit seen with VIM DBS in ET (36). Given the important role the cerebellothalamic tract plays in tremor, the PSA/cZI has been used as an alternative DBS target. A randomized trial comparing the two targets in the treatment of ET, although not statistically significant, favored lower amplitude stimulation of the PSA for tremor control. Owing to the short follow-up duration, any implication on the development of habituation could not be assessed (78). A comparative study comparing the cZI and VIM targets in ET, demonstrated both to be beneficial for tremor suppression; but a potential long-term advantage with applying VIM stimulation due to the gradual worsening of tremor scores in patients stimulated in the cZI region (30). However, other studies have demonstrated persistent long-term benefit with PSA/cZI stimulation and an absence of habituation (33–35). Available evidence suggests PSA/cZI to be equivalent to VIM in effectiveness for tremor suppression but with lower stimulation energy requirements, likely reflecting closer electrode proximity to the dentatorubrothalamic tract. Lower energy requirements for chronic stimulation using the PSA/cZI target could confer some advantage in reducing the risk of habituation but does not appear to eliminate the problem altogether.

PD tremor cohort comparison between VIM-STN and STN-GPi stimulation has occurred with differing strengths of evidence. In a meta-analysis of five randomized control trials, STN and GPi DBS were shown to reduced tremor symptoms without significant differences between the two stimulation targets. STN DBS appeared to reduced tremor severity with a larger effect size in the short-term, while GPi DBS appears to have a steadier and more stable tremor effect in the long-term (62). Further comparison of these two targets, specifically in relationship to action and rest tremor; suggested the initial STN superiority might be due to effective action tremor suppression in the early post-operative period (79). VIM and STN DBS have been compared in a small retrospective analysis, no significant difference in degree of improvement in rest, action or postural tremor was observed (62).

### Is Habituation Different With Unilateral vs. Bilateral VIM DBS?

Despite bilateral VIM DBS leading to a greater overall reduction in tremor severity (80) often owing to the bilateral and midline benefits (18), unilateral VIM DBS has been associated with more persistent tremor benefit from stimulation in ET (12). Favilla et al. (12) demonstrated the benefit from stimulation compared with baseline was consistent through 36-months for both unilateral and bilateral stimulation. However, the loss of delta overtime was 4% in the unilateral compared to 41% in the bilateral group (Table 1). A similar finding was seen in the Cury et al. PD cohort who had undergone VIM DBS, tremor suppression was maintain in both unilateral and bilateral groups through 16–21 years of follow-up, but the loss of delta over time was more pronounced in the bilaterally treated group (29) (Table 1). Both observations may reflect a possible propensity for habituation with bilateral stimulation despite the tremor syndrome. Conceptually, bilateral stimulation could exert greater plastic force on cerebellar networks to adopt abnormal configurations, with less potential for compensation from an untreated side. Other factors that should be considered when counseling patients regarding the possible development of habituation include asymmetry of the tremor syndrome, stimulation intensity and continuous vs. interrupted stimulation.

## Prevention and Management of Habituation to DBS for Tremor

The accuracy of lead placement is critical to avoid early loss of benefit after VIM or PSA/cZI DBS however this problem, by definition, is distinct from true habituation. For patients with well-placed DBS leads possible preventative strategies for habituation include conservative parameter setting to avoid overstimulation and instructing the patient to switch the device off at night (8). Patients may also be advised to only use stimulation the day when needed rather than continuously, in an on-demand fashion, which may reduce the risk of habituation (81). In clinical practice when patients return in long term follow up and report declining tremor benefit, there is a temptation to reprogram usually with increased stimulation current, which often improves tremor but only temporarily. Such increases when performed repeatedly over time may result in chronic DBS-induced ataxic syndrome. An alternate strategy is to refrain from increasing the DBS, clinical worsening is mild and provided patients remain significantly improved compared with the pre-operative baseline.

There are a few studies evaluating varying stimulation programs in an attempt to reduce the occurrence of habitation. Seier and colleagues found reduce benefit decay in patients varying between two equally effective stimulation programs weekly after 12 weeks compared with those receiving unvarying programs (82). However, another study using daily variation of stimulation programs found no superiority to unvarying stimulation at 10 weeks (83). In clinical practice, with limited options available, installing different effective programming groups for patients to vary between is feasible (84), and may reduce habituation, although weekly changes appear more effective than daily. Cycling of stimulation between ON and OFF in blocks of 1–30 s was reported as helpful in reducing habituation and rebound tremor in three PD tremor patients (85).

For patients with established benefit decay and habituation, particularly those with stimulation induced ataxia, reduction of stimulation current may be helpful, and can be more achievable if performed gradually and predominantly unilaterally. Cessation of stimulation for a few days can be attempted, preferably supervised in hospital, and after initial rebound tremor passes, dissipation of DBS induced cerebellar ataxia is expected with a return to the baseline tremor syndrome, and potential functional improvement if ataxia has become the predominant driver for disability. Cessation of stimulation provides an opportunity to reappraise DBS effectiveness in the On vs. Off stimulation condition. Some patients will experience improvement in DBS effectiveness after a period of complete DBS cessation, so-called “stimulation holiday”, however such improvements are usually unsustained but can be repeated in an attempt to recapture benefits lost to habituation (47).

Related to prevention of habituation should be an attempt to stratify the risk of its occurrence in tremor patients being considered for DBS. The tremor subtype is relevant with PD patients less likely to develop habituation than those with ET or DT. Tremor phenomenology may also be useful in predicting risk of habituation, with patients with more action-predominant tremor (7) and those with signs of cerebellar ataxia at greater risk (37).

Surgical approaches to habitation may include reimplantation of DBS with a repositioned electrode optimized to closer proximity to the dentatorubrothalamic tract (as imaged by diffusion tensor tractography MRI) which may achieve superior tremor control with less habituation (86). Moreover, there is growing evidence that closer targeting of MRI-visualized dentatorubrothalamic tract (DRT) provides more effective and efficient tremor control (87, 88) and it remains to be seen whether this approach of targeting DRT deliberately will confer lower rates of habituation in the longer term. Additional “rescue” leads have been implanted in targets including VIM, PSA/cZI, VOA and STN with moderate additional benefit (89, 90). Thalamotomy has been performed after failed VIM DBS including cases with waning benefit and habituation, with modest additional benefit (91).

The advent of more advanced DBS hardware and programming capabilities including independent, constant current directional leads, implanted pulse generators allowing shorter pulse widths <60 μs and sensing of local field potential (LFP) spectra hold promise to assist in the prevention and long-term management of habituation after tremor DBS. Directional leads allow shaped stimulation fields to maximize benefit with fewer side effects, widening the therapeutic window (92, 93). Similarly shorter pulse widths allow greater stimulation current to be delivered without provoking side effects (94, 95). DBS devices allowing real-time recording of LFPs foreshadow closed loop stimulation; the first study demonstrating successful ambulatory recording of VIM and cZI LFPs corresponding to voluntary movements and tremor with highly effective tremor suppression when DBS was delivered closed-loop triggered by LFP activity (96). Of relevance to closed-loop approaches is the important discovery that delivery of DBS stimuli in specific relation to the phase of tremor (phase-locked stimulation) is more effective and efficient with fewer side effects (97).

## Conclusions

Habituation is a real phenomenon after DBS for tremor and is a contributory factor to waning clinical benefit after tremor DBS. The other major contributor to waning benefit is disease progression of the underlying tremor syndrome. Instances of more dramatic loss of clinical benefit over shorter timeframes (short term habituation) may occur and induction of progressive ataxic cerebellar symptoms suggesting aberrant plasticity within cerebellar networks targeted by VIM and PSA DBS. Our current mechanistic understanding of habituation is incomplete and further neurophysiological and imaging studies will be required to elucidate the pathophysiology. In clinical practice, habituation after DBS for tremor remains a feared complication, with available preventative strategies limited to interrupted stimulation regimens (typically switching off at night), minimizing stimulation current or varying programs. It remains to be seen whether technological innovations in DBS such as deliberate MRI-guided targeting of the DRT, directional leads, lower pulse widths or advanced stimulation methods such as phase dependent or on demand DBS will reduce the problem of habituation. In the meantime, it is important that habituation and disease progression of tremor be explained to patients prior to DBS as factors that may result in reduction in clinical benefit over time.

## Author Contributions

JP and ST contributed to the first draft and revision of the manuscript. All authors contributed to the article and approved the submitted version.

## Conflict of Interest

The authors declare that the research was conducted in the absence of any commercial or financial relationships that could be construed as a potential conflict of interest.

## Publisher's Note

All claims expressed in this article are solely those of the authors and do not necessarily represent those of their affiliated organizations, or those of the publisher, the editors and the reviewers. Any product that may be evaluated in this article, or claim that may be made by its manufacturer, is not guaranteed or endorsed by the publisher.
